# Network effects on coordination in asymmetric games

**DOI:** 10.1038/s41598-017-16982-2

**Published:** 2017-12-05

**Authors:** Joris Broere, Vincent Buskens, Jeroen Weesie, Henk Stoof

**Affiliations:** 1 Utrecht University, Department of Sociology/ICS, Utrecht, The Netherlands; 2 Utrecht University, Institute for Theoretical Physics, Utrecht, The Netherlands

## Abstract

Network structure can have an important effect on the behavior of players in an iterated 2 × 2 game. We study the effect of network structure on global and local behavior in asymmetric coordination games using best response dynamics. We find that global behavior is highly dependent on network topology. Random (Erdös-Rényi) networks mostly converge to homogeneous behavior, but the higher the clustering in the network the more heterogeneous the behavior becomes. Behavior within the communities of the network is almost exclusively homogeneous. The findings suggest that clustering of networks facilitates self-organization of uniform behavior within clusters, but heterogeneous behavior between clusters. At the local level we find that some nodes are more important in determining the equilibrium behavior than other nodes. Degree centrality is for most networks the main predictor for the behavior and nodes with an even degree have an advantage over nodes with an uneven degree in dictating the behavior. We conclude that the behavior is difficult to predict for (Erdös-Rényi) networks and that the network imposes the behavior as a function of clustering and degree heterogeneity in other networks.

## Introduction

Coordinating interdependent behavior when actors have different interests can be extremely difficult. These types of situations can be represented by an asymmetric ‘Battle of the Sexes’ game as shown in Table 1. Table 1 represents a situation in which two actors have a choice between *α* and *β*. Coordination on the same choice yields the highest payoff, but the actors differ in their preference for either *α* or *β*. Analyzing this situation, it becomes clear that the Nash equilibria seem either unfair, because one actor is better off than the other, or inefficient in the case of a mixed Nash equilibrium^1^. Therefore, it is difficult to make behavioral predictions on the choices of players in these types of situations. The coordination problem is potentially larger when there are more than two actors involved. However, in a three player game, the behavior is already easier to predict when two players have a preference for *α* and one has a preference for *β*. The most likely Nash equilibrium is for all players to play *α*, because this yields the highest utility for the two *α* players, independent of what the *β* player does. In games with more than three players, the behavioral prediction is dependent on the interaction structure. When the interaction structure is represented as a network, there are local majorities of actors that have a preference for one of the equilibria. Therefore, it seems plausible that the network structure is crucial for understanding an asymmetric coordination problem with multiple actors. In this paper we study the influence of network structure on global and local behavior in iterated asymmetric coordination games.

**Table 1:** Payoff table of an asymmetric coordination game.

	*α*	*β*
*α*	2, 1	0, 0
*β*	0, 0	1, 2

Situations in which interdependent actors have to coordinate behavior with unequal preferences are wide-spread. The classic 2 by 2 example is the situation of a man and a woman who have to coordinate their evening out without means of communication. The man has a preference to go to a football match and the woman prefers to go to the opera, however, they still prefer to go to the same event over going to the events alone. It is very hard to coordinate this problem with no means of communication. There are also contexts in which the group and interaction structures are relevant. Think for instance about a school class in a theme park that has to decide which attraction to go to next. Some of the children prefer to go to the roller coaster and others prefer to go to the water attraction. However, they also prefer going with their friends instead of going alone. It is therefore likely that a person with a preference for attraction *A* will still go to attraction *B* if the majority of his or her friends have a preference of attraction *B*. So, whether a preferred option is also the option which yields the highest utility might be dependent on the preferences of others in the network and the interaction structure. Similar dynamics can be expected when people have to choose between (operating) systems on their phone, computer or game consoles. For some applications it is necessary that your friends have the same system, so coordinating on the same system as your friends might yield more utility than choosing your personal favorite system.

A classical game theoretical example of modeling asymmetric situations is the so-called ‘Battle of the Sexes’ (BoS). BoS is a special case of a coordination game with two pure-strategy asymmetric equilibria and one mixed strategy equilibrium. As presented in Table 1, the players differ with respect to their preferences over the equilibria, but coordinating on the same equilibrium is still preferred over miscoordination. The equilibria of this game have been widely studied theoretically and empirically in both one shot and repeated versions of BoS^1,2^. However, it is not at all clear for the network version of BoS to what equilibrium the game will converge, if at all. Emerging equilibrium behavior is likely dependent on the initial conditions and the spatial structure of the network.

Some research has been done on ‘Battle of the Sexes’ types of games in the context of spatially distributed interactions in both theoretical and experimental settings. A great amount of work on spatial BoS games is done by Alonso-Sanz^3–5^, mostly in the context of homogeneous spatial structures such as cellular automata. Some interesting results include the ability of self organization by means of forming homogeneous clusters in the spatial structure. Furthermore, Hernandez, Muñoz-Herrera and Sánchez^6^ introduce a model to analyze the Nash equilibria of an asymmetric game on a network. They find that on Erdös-Rényi networks equilibria exists where all players can choose their preferred action when the heterogeneity in the network is high, whereas players tend to coordinate on the other action when they are in a clear minority situation. In related work the influence of the strength of preferences is studied^7^. The higher the difference in preferences the harder it is to reach coordination. However, when the difference in the preferences are small, full coordination is always an equilibrium. Mäs and Nax^8^ studied the response behavior of human subjects in two fixed networked coordination games in an experimental setting. An interesting finding is that 96 percent of the decisions followed a myopic best response pattern. Other related studies show the effect of asymmetries on cooperation in (weak) Prisoner’s Dilemma games^9–11^. An important result is that asymmetry introduced by payoff heterogeneity or mixed games has a favorable effect on cooperation. The main difference between our study and the studies above is that we study the effect of network structure on behavior, both on the local and global level and we derive predictions from the network structure.

To the best of our knowledge, no studies have been performed on the influence of the spatial structure of a network on the equilibrium behavior in a BoS game. We believe that studying a BoS game is particularly interesting because these games provide us with information on which types of nodes end up in their preferred equilibrium and which do not, dependent on the spatial position. If network structure is of any influence, some nodes should have more powerful positions in the sense that in their position in the network they more easily coordinates on its preferred behavior. Therefore, network structure can be of vital importance in understanding asymmetric coordination problems with multiple actors.

Network structure is often found to be a crucial concept in understanding many phenomena such as virus spreading, percolation, social cooperation and information diffusion^12–15^. Many studies have shown that differences in network topology can lead to wildly different behavior. These studies often focus on global network effects, such as cooperation in a Prisoner’s Dilemma game, or just local effects, such as identifying influential spreaders in diffusion models^16–18^. We argue that global and local dynamics are inevitably dependent on each other. Considering that network structure has an effect on global level dynamics, there must be an effect on the local level as well and vice versa.

Game theory is an often used method of modeling network dynamics^12,13^. Game theory is a set of analytical tools to represent interdependent situations between agents, designed to model and make predictions on decision making. In the networked game, agents play games with a subset of a population, represented by edges in a network. Many computational studies have shown that the spatial structure of a network can have an influence on the evolution of behavior in a game^12,19,20^. Santos *et al*.^16^ compare complete, small-world and scale-free networks on cooperative behavior. They show that heterogeneity of the degree distribution has an important influence on the asymptotic density of cooperators in symmetric games such as Stag-Hunt and Prisoner’s Dilemmas. Tomassini and Pestelacci^21^ show that the presence of clusters of highly connected nodes in a network has a positive impact on cooperation. However, too much clustering can lead to so-called ‘topological traps’^22^. These traps can prevent cooperation from spreading uniformly through the network. Most of these studies focus on the effect of the spatial structure on the behavior of symmetric games, such as symmetric Prisoner’s Dilemmas and symmetric coordination games. However, interesting situations can also arise in asymmetric games.

## Methods

We utilize a ‘Battle of the Sexes’ coordination game to represent actors with their different preferences. Assuming that the behavior of the most influential or powerful network positions are more likely to converge to their preferred equilibrium than the less influential or powerful network positions, spatial effects can be understood by the probability of a given node in a network to end up in its preferred equilibrium, irrespective of other initial conditions.

In Table 2a the utility matrix of the 2 × 2 BoS game is presented. The game is modeled such that 0 < *S* < 1. In this case the row player has the highest payoff when both players choose *α* and the column player has the highest payoff when both players choose *β*. Coordinating on the same behavior is more rewarding than miscoordination. The 2 × 2 game can be mapped on a network by pairwise interactions between nodes who share an edge. In this network, every node has a preference for either *α* or *β*, so is a row or a column player. When nodes play pairwise interactions against multiple nodes on a network, three situations can occur. The first situation is a pairwise interaction between two nodes who differ on their preference for *α* or *β*, as already described in Table 1 and again in Table 2a. In the second situation, two nodes with the same preference for *α* pairwise interact, as shown in Table 2b. In the third situation, two nodes with the same preference for *β* interact, as shown in Table 2c. In the 2 by 2 case, coordination is difficult in the first situation, but rather obvious in the second and third situation.

**Table 2:** Possible payoff situations in BoS on a network, where 0 < *S* < 1.

	(a)			(b)			(c)	
	*α*	*β*		*α*	*β*		*α*	*β*
*α*	1, *S*	0, 0	*α*	1, 1	0, 0	*α*	*S*, *S*	0, 0
*β*	0, 0	*S*, 1	*β*	0, 0	*S*, *S*	*β*	0, 0	1, 1

A potential extra difficulty is added when nodes have to coordinate with multiple nodes at the same time in a network, while having to choose for each connection the same behavior^23^. The total utility of a node depends on the returns of multiple interactions at the same time. Say node *i* has *d* neighbors and a preference for playing *α*. A fraction *q* of the neighbors play *α* and the fraction (1 − *q*) plays *β*. If node *i* chooses *α* the payoff will be *qd* as the utility will be 0 for miscoordinating with nodes who play *β*. If node *i* chooses *β* the payoff will be (1 − *q*)*dS*. Assuming node *i* knows *q*, node *i* will choose *α* if

1 $$qd\ge \mathrm{(1}-q)dS\mathrm{.}$$

By rearranging the terms,

2 $$q\ge \frac{S}{1+S},$$

it becomes clear that the choice for *α* or *β* is dependent on the fraction of neighbors that play *α* or *β*. However, the game is played multiple rounds and the nodes don’t know in advance what the strategy is that their neighboring nodes will play.

In each round the nodes update their belief on what strategy yields the highest payoff, *α* or *β*, by means probabilistic dynamic in which the behavioral propensity changes towards the best response. Let *i* = 1 … *N* be the nodes in the population. Let *s* ∈ {*α*, *β*} be the strategy of node *i*, *π*_*i*_ the payoff of node *i* and ${\pi }_{i}^{^{\prime} }$ the payoff when the alternative strategy would have been played. Then, the probability ${p}_{s,i}^{t}$ that a strategy *s* is played in round *t*, given the probability ${p}_{s,i}^{t-1}$ that a strategy *s* is played in round *t* − 1 equals;

3 $${p}_{s,i}^{t}=\{\begin{array}{ll}{p}_{s,i}^{t-1}+0.1 & {\rm{for}}\,{\pi }_{i}^{t-1}\ge {\pi }_{i}^{^{\prime} ,t-1}\\ {p}_{s,i}^{t-1}-0.1 & {\rm{for}}\,{\pi }_{i}^{t-1} < {\pi }_{i}^{^{\prime} ,t-1},\end{array}$$

where ${p}_{s,i}^{t}$ is the probability that strategy *s* is played at some time *t* by node *i* ^20^. So, at every time *t* each node updates the probability to play *α* or *β* towards a myopic best response reply strategy. If the best reply at time *t* − 1 would have been *α*, the probability of playing *α* at time *t* increases compared to time *t* − 1. If the best reply at time *t* − 1 would have been *β*, the probability of playing *α* at time *t* decreases at time *t* (simultaneously the probability to play *β* increases). The probabilities are naturally bounded by the values 0 and 1.

Various studies have shown that the results of a spatial game may depend on the update rule implemented^24–27^. We choose this probabilistic response behavior because the dynamics gradually move towards the best response and therefore quickly converges to an evolutionary stable state. We also compared the results to the deterministic version of myopic best response. Some results with the deterministic myopic best response can be found in the Fig. S1 of the supplementary materials of this paper. Although the substantive results stay the same with the deterministic update rule, this update rule is not suitable because a large portion of networks do not converge due to eternal state switching between nodes. Another common version of myopic best response, as implemented by Szolnoki & Perc and Rong *et al*.^24–26^, can be considered a version closer to the deterministic or the probabilistic update rule implemented in this study, depending on the value chosen for the uncertainty parameter. Given the robustness of our findings depending on the deterministic or probabilistic-update rule, we also don’t expect any substantive differences with such other update rule.

### Simulation Design

We perform a computational study in which actors play 2 × 2 games against their neighbors represented by the nodes and edges of a network. Three types of networks are considered, namely random Erdös-Rényi (ER) networks, small-world (SW) networks, and preferential attachment (PA) networks. The ER-networks are generated using the *G*(*N*, *p*_*er*_) Erdös-Rényi model, where *N* is the number of nodes in the graph and *p*_*er*_ the probability for drawing an edge between two arbitrary nodes. We choose *N* = 20 and *p*_*er*_ = 0.2 in our simulation. The SW-networks are generated using the Watts-Strogatz algorithm^28^. The algorithm starts with a one-dimensional lattice consisting of *N* = 20 nodes. Each node is connected with two neighboring nodes by an edge. The edges are rewired randomly with probability *p*_*sm*_. SW-networks are known to have short average path lengths and high clustering. The clustering decreases with the value of *p*_*sm*_. The higher the value of *p*_*sm*_, the more the network will resemble the Erdös-Rényi model in terms of clustering^28^. In order to vary the amount of clustering within SW-networks, we vary *p*_*sm*_, where *p*_*sm*_ ∈ {0.05, 0.1, 0.15, 0.2, 0.25}. The PA-networks are generated by the algorithm proposed by Barabasi and Albert^29^. The algorithm starts with *m*_0_ nodes. With each iteration one new node with *m* edges adds on to the existing nodes with probability *p*_*pa*_, where ${p}_{pa,i}={k}_{i}/{\sum }_{j}\,{k}_{j}$, and where *k*_*i*_ is the degree of node *i* and the sum is made over the previously added nodes. This process continuous until the network consists of *N* = 20 nodes. We choose *m* = 2 in order to keep the density of the network equal to the other types of networks. We choose *N* = 20 for all networks in this study because with this size the relative influence of one node on the global behavior can still be substantial, while this size is big enough to guarantee the complexity of behavior in the network. Results for larger network sizes will be discussed under the robustness analyses while the related graphs can be found in the supplementary materials of this paper. We only include connected graphs for all network types throughout the whole simulation.

All networks are generated with size 20 of which 10 nodes have a preference for *α* (row players in Table 2a) and 10 nodes have a preference for *β* (column players in Table 2a). The constraint of 10 *α* and 10 *β* players is imposed to maximize the coordination problem. The preferences of the nodes are randomly assigned. In the simulation a 1000 ER-networks, SW-networks and PA-networks are generated, thus 3000 networks in total. For each network the game is played 100 times.

After the networks are initialized the iterated game starts. In each iteration a node has to choose between *α* and *β*. The total obtained utility is a function of the actions of the neighboring nodes, given by the utility matrix in Table 2. We chose *S* ∈ {0.9, 0.7, 0.5}. If *S* becomes smaller, the incentive to deviate from one’s preferred behavior becomes smaller as well. In the remainder of this paper we only discuss results for the single case *S* = 0.9 because this value maximizes the dynamics in the system. Results for other values of *S* can be found in the supplementary materials of this paper. Initially, at *t* = 0, each node plays its preferred option with probability 1. After each round the probabilities to play *α* or *β* are updated by means of the response decision rule described in Equation 3. The game continues until none of the nodes changes probabilities anymore, so all probabilities are either 0 or 1 to play *α*. The iterations run with a minimum of 10 and a maximum of 100 iterations. After each game several variables are saved. These include the initial conditions, several node and network characteristics and the equilibrium state of the node and the network. All files necessary to replicate the simulation can be found on the first author’s github page: https://github.com/JJBroere/Network-effects-on-coordination-in-asymmetric-games.

### Variables

The goal of this study is to infer how equilibrium behavior depends on the initial conditions of the networks as well as the power of an individual node to determine its equilibrium behavior given its position in the network. The first dependent network level variable is the proportion of nodes playing *α* in the network. A proportion of 1 indicates that all nodes in the network play *α* and a proportion of 0 indicates all nodes in the network play *β*. We define heterogeneity of the behavior as the variance of *α* behavior in the network;

4 $$h({p}_{\alpha })={\rm{v}}{\rm{a}}{\rm{r}}({p}_{\alpha })={p}_{\alpha }(1-{p}_{\alpha }),$$

where *p*_α_ is the proportion of nodes playing *α* in the network.

The second dependent variable is the dichotomous variable *Preferred*, indicating whether a node ends up in the equilibrium of preference after the game has converged. Third, we define *Power* of a node as the probability of a node to converge to its preferred equilibrium independent of the distribution of preferences. So, if different distributions of preferences on a network are played, what is the proportion of times a node converges to the preferred equilibrium given its position in the network. 100 different distributions of preferences per network will be played. *Power* is thus, the aggregate variable of *Preferred*.

In addition, we consider several independent variables that could be indicative of the equilibrium behavior of the game. The first set of independent variables are centrality measures. From common sense it might be expected that the most central nodes in a network are the most powerful. A wide range of centrality measures have been developed to account for different aspects of centrality in a network. In this paper four different centrality measures are considered, namely degree centrality, eigenvector centrality, betweenness centrality and closeness centrality. See the supplementary methods section in the supplementary materials of this paper for the formal definitions of the centrality measures used in this paper. Furthermore, we add a dummy variable indicating whether the degree centrality of a node is even or uneven. As can be derived from Equation 2, where *S* = 0.9, the fraction of neighbors playing the preferred behavior of node *i* in order for node *i* to also play its preferred behavior is different for nodes with an even degree compared to an uneven degree. As presented in Table 3, the fraction where *q* > *S*/(1 + *S*), thus *q* > 0.474 is always 50% for nodes with an even degree centrality while the fraction is bigger for an uneven degree centrality. The difference decreases as the degree centrality increases.

**Table 3:** Number of neighboring nodes required for a local majority.

Degree centrality	1	2	3	4	5	6	7	8
Number of neighboring nodes needed	1	1	2	2	3	3	4	4
Percentage of neighboring nodes needed	100%	50%	67%	50%	60%	50%	57%	50%

Secondly, we look at the influence of clustering on the behavior in the network. Clusters, modules or communities are closely connected subgraphs within a network. As shown by Roca *et al*.^22^, clustering can be an important predictor of whether or not behavior will spread uniformly through a network. Alonso-Sanz^3^ shows how behavior self-organizes in homogeneous (agreement) clusters when played on cellular automata. We expect that the self-organizing behavior of preferences will be influenced by network characteristics and more specifically the clustering of the network. This clustering effect might be even more relevant when groups of nodes with the same preference are clustered together.

In this study we use the Walktrap algorithm as described by Pons and Latapy^30^ to identify communities in a network. This algorithm performs a random walk on the graph. The main idea is that in clustered networks the random walk is more likely to remain in the same community than leave the community because of higher connectedness within the community. For every node in the network the random walk is used to compute a ‘distance’ between all pairs of nodes. The computed distance is used to find the partition that maximizes the modularity.

We also use the modularity to quantify how clustered a network is. Modularity is the fraction of edges within one module minus the expected fraction of edges if the edges where distributed at random^31^. Modularity of a network is defined as;

5 $$Q=\frac{1}{2N}\,\sum _{ij}\,[{A}_{ij}-\frac{{k}_{i}{k}_{j}}{2N}]\,\delta ({c}_{i},{c}_{j}),$$

where *N* is the number of nodes, *A* is the adjacency matrix, *k* the degree of a node, *c* is the number of the community a node belongs to as identified by the Walktrap algorithm described above and *δ*(*x*, *y*) is 1 if *x* = *y* and 0 otherwise.

The third set of independent variables are related to the initial conditions of the network. The preferences of the nodes are randomly assigned on the network. In order to evaluate how the equilibrium behavior is related to the local distribution of preferences, for each node two variables are saved. First, for every node, the percentage of neighbors with the same preference is saved, defined as;

6 $${L}_{i}^{n}=\frac{{\sum }_{j}{A}_{ij}\delta (p{r}_{i},p{r}_{j})}{{\sum }_{j}{A}_{ij}},$$

where *A* is the adjacency matrix, *pr* is the preference of the node (row or column player).

Secondly, the percentage of nodes with the same preference within the same community is saved, defined as;

7 $${L}_{i}^{c}=\frac{{\sum }_{j}\delta ({c}_{i},{c}_{j})\,\delta (p{r}_{i},p{r}_{j})}{{\sum }_{j}\delta ({c}_{i},{c}_{j})},$$

where *c* is the community a node belongs to as identified by the Walktrap algorithm described above.

### Descriptive statistics

In Table 4 the descriptive statistics of all dependent and independent variables are presented. The dataset contains of 3,000 networks, information about 20 nodes and 100 starting configurations per network, leading to 6,000,000 ‘observations’. S3 in the supplementary material of this paper shows that the variance of the variable *Preferred* caused by the stochasticity of the response dynamics is limited when the same preference distribution is played on a network multiple times. S4 of the supplementary materials shows that the estimates of *Power* are stabilized after 100 different initial conditions.

**Table 4:** Descriptives of the dependent and independent variables.

Statistic	N	Mean	St. Dev.	Min	Max
Proportion *α*	300,000	0.500	0.337	0	1
Heterogeneity	300,000	0.146	0.104	0	0.250
Preferred	6,000,000	0.640	0.478	0	1
Power	60,000	0.640	0.108	0.2	1
Eigenvector centrality	60,000	0.523	0.245	0.004	1
Betweenness centrality	60,000	0.101	0.108	0	1
Degree centrality	60,000	0.191	0.115	0	1
Same preference cluster	60,000	0.471	0.254	0	1
Same preference neighbors	60,000	0.474	0.322	0	1
Modularity	3,000	0.313	0.069	0.112	0.518

## Results

**Figure 1:**
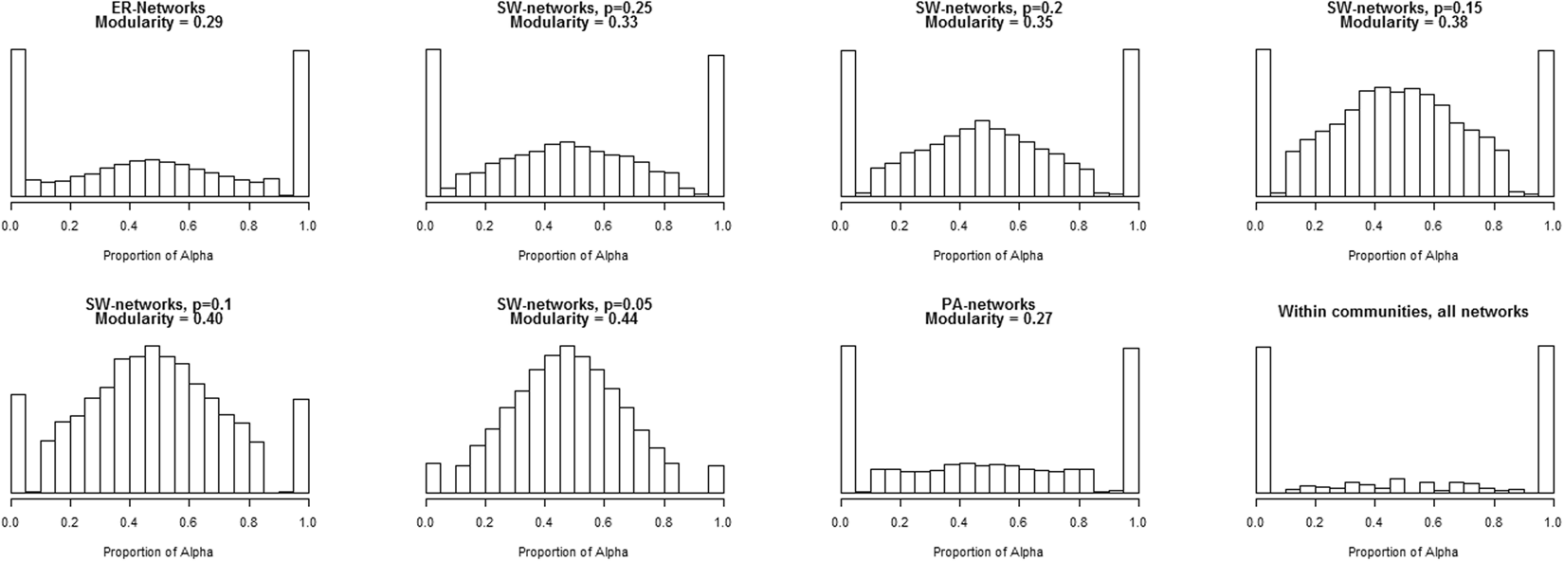
Proportion of *α* played in a network after convergence for ER-networks, SW-networks with rewiring probability 0.25, SW-networks with rewiring probability 0.2, SW-networks with rewiring probability 0.15, SW-networks with rewiring probability 0.1, SW-networks with rewiring probability 0.05, PA-networks and within communities of all networks.

To get a first impression on how the equilibrium behavior is related to network type we first look at the global behavior of the networks. In Fig. 1 histograms are shown for the proportion of *α* behavior after convergence of the dynamics. As can be seen in Fig. 1, most frequently the behavior in ER-networks converges to either uniform *α* or uniform *β* behavior. The behavior for SW-networks are shown for different values of the rewiring probability *p*_*sm*_. When *p*_*sm*_ = 0.25 the behavior is mostly uniform *α* behavior or uniform *β* behavior, closely resembling the outcome of the ER-Network. However, the lower the value of *p*_*sm*_, and therefore the higher the level of modularity/clustering, the more heterogeneous the behavior becomes. The behavior in SW-networks with *p*_*sm*_ = 0.05 seldom converges to uniform behavior. PA-networks have a low modularity and the behavior is mostly homogeneous. This trend can be described by the Pearson correlation between the heterogeneity of behavior in the network and the modularity of the network, which is *r* = 0.49. In the bottom right histogram of Fig. 1 the behavior is shown within communities of all networks in the data. The convergence behavior within communities of networks is almost exclusively homogeneous in all networks.

**Figure 2:**
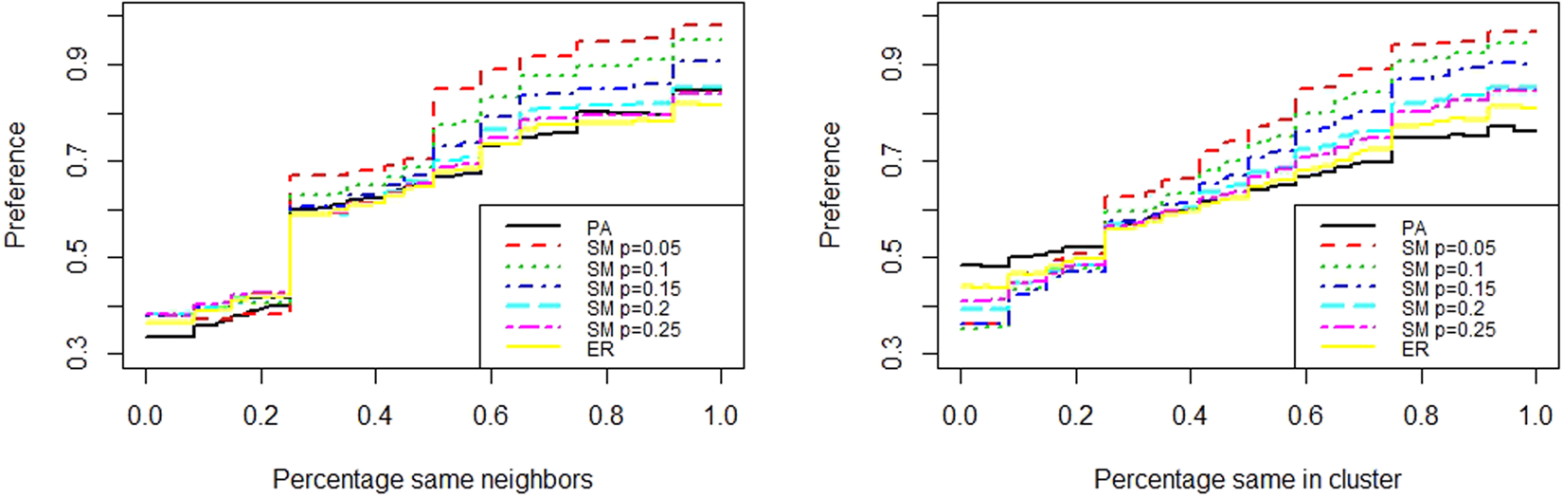
Kernel regression plot, dependent variable *preference* as a function of, left the fraction of same preference neighbors, right the fraction of same preference nodes in the community.

In Fig. 2, kernel regression plots are shown for the dependent variable *Preferred*. Kernel regression is a non-parametric technique of estimating the conditional expectation of a random variable. The conditional expectation is computed by a locally weighted average given some kernel as a weight function. The kernels in Fig. 2 are estimated with a box kernel and bandwidth = 0.5. The two predictors are the proportion of adjacent neighbors that have the same preference as shown in the left part of Fig. 2 and the proportion of nodes that have the same preference in the same community as shown in the right part of Fig. 2. The plots indicate a strong relation between the clustering of the preferences and the expected probability that a node behavior converges to its preferred behavior. There seems to be a clear tipping point for the effect of the percentage of neighboring nodes with the same preference where there is a local majority of preferences. This relation clearly is stronger for the more clustered SW-networks compared to the ER-networks and PA-networks, indicating that the local interactions become more important in more clustered networks.

**Figure 3:**
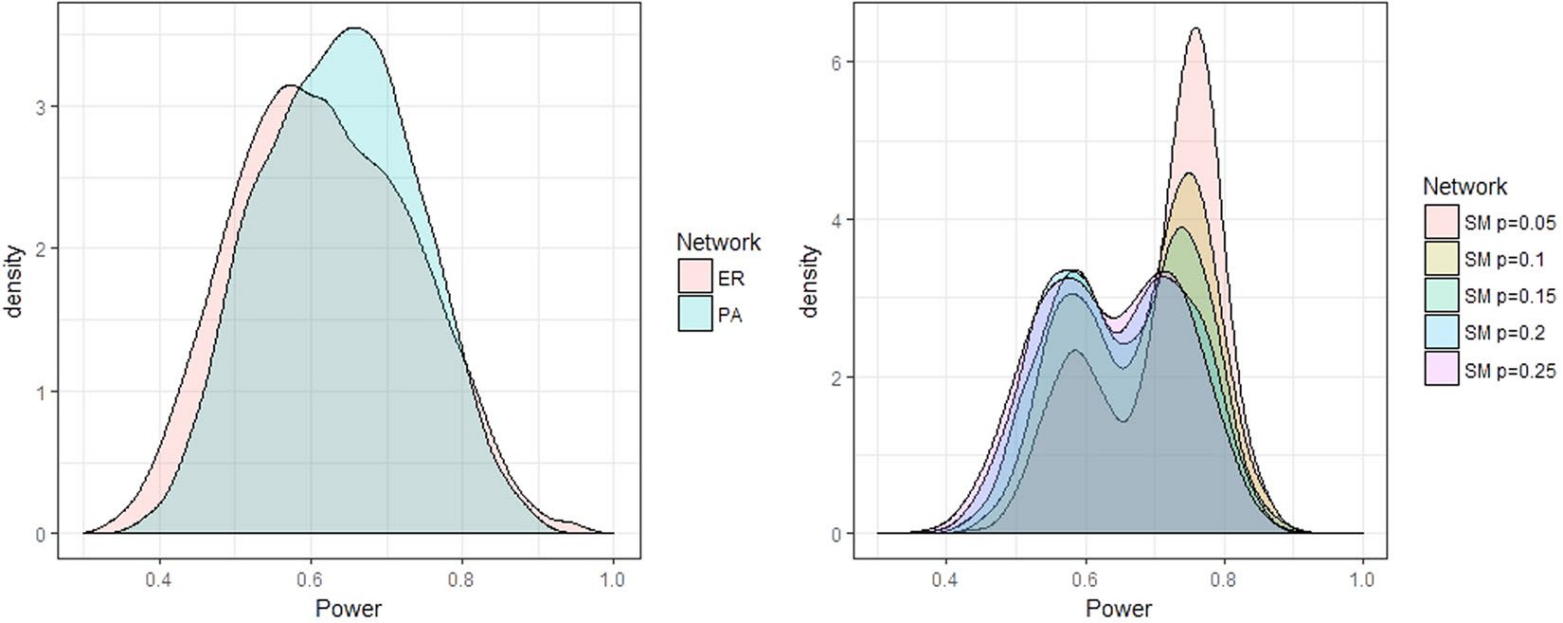
Density of node *Power* for random ER, Small World and PA-networks.

In Fig. 3 the density of the variable *Power* is plotted for ER, SW and PA-networks. The higher the proportion the more power a node has to determine its equilibrium behavior given its position in the network. A proportion of 0.5 indicates that the behavior is random and there is no association between the nodes spatial position and its equilibrium behavior. If the proportion is 1, the node always converges to the preferred equilibrium and has maximum power to determine its equilibrium behavior. As can be seen in the left part of Fig. 3, for ER-networks the density roughly follows a normal distribution with a mean of 0.618. The density of *Power* in PA-networks is comparable to the density of ER-networks with a mean of 0.637. On the right of Fig. 3 the densities of SW-networks for different values of *p*_*sm*_ are plotted. The lower the value of *p*_*sm*_ the more the density shifts to the right, indicating that there are more powerful nodes in SW-networks with lower value of *p*_*sm*_. Because more clustered networks have more heterogeneous behavior, more nodes will be able to choose their preferred behavior.

In Table 5 the OLS-regression results with node *Power* as dependent variable are presented. Interactions between all variables are included in the model. To obtain the relevant predictors we used cross validated backward model selection based on a minimum difference of 0.01 in the *R* ^2^ of the model. There are two types of variance in the data, namely the within network and the between network variance. As shown in Table 5 the inter class correlation (ICC) does not exceed 0.022 for SW, is 0.062 for PA-networks and is 0.091 for ER-networks. We decided to ignore the between-level variance since most of the variance is on the within-level. The explained variance is 62.8 percent for ER-networks, 62.2 percent for PA-networks and always more than 70 percent for SW-networks.

**Table 5:** Regression results, standardized, dependent variable *Power*.

	ER	SW *p* = 0.25	SW *p* = 0.20	SW *p* = 0.15	SW *p* = 0.1	SW *p* = 0.05	PA
Even	0.154	0.159	0.160	0.165	0.165	0.172	0.155
DegC		0.210	0.197	0.191	0.166	0.155	0.344
EVC	0.025						
BetC	0.964						
ClosC	−0.470						
BetC:EVC	−0.839						
EVC:ClosC	0.369						
Constant	0.627	0.478	0.488	0.499	0.516	0.524	0.490
N	20,000	4,000	4,000	4,000	4,000	4,000	20,000
R^2^	0.628	0.726	0.714	0.739	0.740	0.757	0.532

Even = variable indicating an even degree, EVC = Eigenvector centrality, BetC = Betweenness centrality, DegC = Degree centrality, ClosC = Closeness centrality. Interaction in uncentered variables.

Regression results indicate that an *Even* degree centrality is an important predictor for node *Power* in all types of networks. For all networks the predicted score for a nodes power increases around 0.16, indicating a 16 percent higher probability to converge to its preferred behavior when a node has an even degree centrality. For nodes with an even degree it’s easier to obtain a local majority compared to nodes with an uneven degree as shown in Table 3. For PA-networks there is one other important predictor for node *Power*, namely degree centrality. Nodes with the highest degree centrality have a 34.4 percent higher predicted probability of percent to converge to its preferred behavior. Nodes with an even degree and a high degree centrality the predicted probability is 98.9 percent. This seems to indicate that the global behavior is predominantly dictated by a few influential nodes with high degree centrality. In SW-networks degree centrality is also an important predictor for node *Power*, the effect is weaker compared to PA-networks. The final model for ER-networks is a complicated model in which all centrality measures are important predictors and the model has difficult to interpret interactions. But it is clear that in ER-networks having a high degree is not the only centrality measure leading to high degree centrality.

All results together seem to indicate that in ER-networks the global behavior is mostly homogeneous, but difficult to predict since this behavior is dependent on multiple centrality measures at the same time. In PA-networks the behavior is also mostly homogeneous, however in this type of network the behavior is dictated by a few influential nodes with high degree centrality. In SW-networks with high clustering, degree centrality is also important, but the spread of behavior is limited by the a node’s community, leading to heterogeneous global behavior. In all networks, nodes with an even degree have an advantage over nodes with an uneven degree, because it is easier for nodes with an even degree to obtain a local majority.

### Robustness

In order to check the robustness of the results we also varied the network size, the network density and the value of *S*. The results can be found in the supplementary materials of this paper. As can be seen in Fig. S5, more clustered networks have more heterogeneous behavior also in larger networks, the differences become even stronger for larger networks. As can be seen in Figs S6 and S7, when network density increases the heterogeneity of behavior becomes less because the modularity decreases as the density increases. As can be seen in Figs S8 and S9, there is a dependency at the local level on the size of the network. First of all, the positive effect of an even degree centrality decreases as the average degree centrality increases, which can be expected by looking at Fig. 3. Degree centrality is the main predictor in SW-networks and PA-networks, however the relative *power* of one node decreases in larger networks, as can be seen from the decreasing regression coefficients. Secondly, in larger networks with high clustering, centrality measures are no longer predictive of node *power*, because the centrality measures are with respect to the network as a whole, while the spread of behavior is limited by the clustering. In this case the position of a node within its community is probably more important than its position in the network as a whole. In Figs S10 until S13 some results for the values of *S* = 0.7 and *S* = 0.5 are shown. For *S* = 0.7 the results are comparable to those of *S* = 0.9. For *S* = 0.5 nodes are less likely to change their behavior, because the fraction *q* = 0.5/(1 + 0.5) = 0.33 is lower. This can be seen in the higher intercepts of the regression models and the lower effect of the predictors. However, it should be noted that even with *S* = 0.5 the behavior within clusters is mostly homogeneous.

## Discussion

In this paper we study the effect of network structure on global equilibrium behavior and the behavior of individual nodes in asymmetric ‘Battle of the Sexes’ games. Looking at the heterogeneity of behavior in the network, we find that network topology has a large effect on the global equilibrium behavior of the networks. The heterogeneity of behavior is largely determined by the modularity of the network. These findings are independent of network size and even visible in other values of *S*. When there is a clear community structure in the network, coordinating on the same behavior within the community is more rewarding than coordinating with nodes outside the community, simply because a node has on average more edges in its community than outside its community. So, in networks with more clearly defined communities (high modularity), some communities can coordinate on one behavior while other communities coordinate on the other behavior, leading to more heterogeneity in behavior at the global level. The ratio of edges inside and outside the community is lower for networks with low modularity, so edges inside the community are almost equally important as edges outside the community. Therefore, nodes often coordinate on the same behavior throughout the whole network in networks with low modularity, in particular in ER-networks.

The same mechanism of clustering is at play when we look at the effect of the initial conditions. Whether a node converges to the preferred equilibrium is largely dependent on the preferences of the neighboring nodes. The probability that a node ends up in the preferred equilibrium can largely be predicted by the majority of preferences in the adjacent nodes or the nodes in the community the node belongs to. This relation is much stronger for more clustered networks. Clustering makes the relation to the direct neighbors and nodes within a cluster more important than the relation outside of the cluster. Together with the finding that high modularity limits the spread of uniform behavior, it can be expected that at some value of modularity the cluster topology becomes more important than the global level topology for the equilibrium behavior.

However, determining what the actual equilibrium behavior will be is not as simple as calculating the majority of preferences in a community. Not every node has the same weight or power to determine the behavior. Nodes with an even number of adjacent nodes have an advantage over nodes with an uneven number of adjacent nodes. A node with three neighbors needs two out of three to choose the same behavior, while a node with four neighbors needs two out of four neighboring nodes to choose the same behavior. Also, degree centrality plays an important role. PA-networks have low clustering and the degree distribution is known to have many nodes with low degree centrality and a few with high degree centrality. Therefore, the global behavior is mostly determined by a few nodes with high degree centrality. In SW-networks degree centrality is also important, but because of its clustering, the influence of a node with high degree on the global behavior is limited to the behavior of the community the node belongs to. For ER-networks global behavior depends in a less clear way on individual network positions. Because the clustering is low and degree distribution is less heterogeneous than PA-networks, often more complex and random network characteristics determine to which behavior the network converges.

It will be interesting if future research focuses on how the cluster (or meso) level is related to the local (or micro) level and global (or macro) level behavior. So, at what value of modularity is the meso-level topology more important for the dynamics than the global topology? In future research, we plan to test these computational results empirically on human subjects in an experimental study.

## Electronic supplementary material

Supplementary Materials
