# Supplementary material for: Network effects on coordination in asymmetric games
Source: Sci Rep. 2017 Dec 5;7:17016. doi: 10.1038/s41598-017-16982-2 (PMC5717250; doi:10.1038/s41598-017-16982-2)
Supplement: Supplementary file 1 — Supplementary Materials [file 41598_2017_16982_MOESM1_ESM.pdf]

Network effects on coordination in asymmetric games
Supplementary materials

Joris Broere[1], Vincent Buskens[1], Jeroen Weesie[1], Henk Stoof[2]

[1]) Utrecht University, Department of Sociology/ICS, Utrecht, the Netherlands
j.j.broere@uu.nl
[2]) Utrecht University, Institute for Theoretical Physics, Utrecht, the Netherlands

S1: Regression results with deterministic myopic best response

Table 1: Regression results, standardized, dependent variable *Power*

	ER	SW $p = 0.25$	SW $p = 0.20$	SW $p = 0.15$	SW $p = 0.1$	SW $p = 0.05$	PA
Even	0.139	0.138	0.140	0.143	0.137	0.141	0.138
DegC		0.268	0.251	0.187	0.160	0.162	0.329
EVC	0.114						
BetC	0.428						
ClosC	−10.997						
Constant	0.829	0.605	0.611	0.612	0.609	0.592	0.586
N	20,000	4,000	4,000	4,000	4,000	4,000	20,000
R^2	0.608	0.666	0.658	0.668	0.638	0.620	0.642

**Even = variable indicating an even degree, EVC= Eigenvector centrality, BetC= Betweenness centrality, DegC = Degree centrality, ClosC= Closeness centrality. *Interaction in uncentered variables.*

S2: Supplementary methods, Centrality measures

Degree centrality is the number of edges a node has. For a given graph $G := (V, E)$ comprising a set V of nodes together with a set E of edges, let $\boldsymbol{A}$ be the adjacency matrix, i.e. $(A_{ij}) = 1$ if node i is linked to node j, and $(A_{ij}) = 0$ otherwise. Then the degree centrality is defined as:

$$k_i = \sum_j A_{ij}. \tag{1}$$

Eigenvector centrality is the centrality score of a node weighted for the centrality scores of the neighboring nodes. The relative centrality score of a node can be defined as:

$$x_i = \frac{1}{\lambda} \sum_{j \in G} A_{ij} x_j, \tag{2}$$

where λ is a constant. With a rearrangement this can be written in vector notation as the eigenvector equation:

$$\boldsymbol{Ax = \lambda x,} \tag{3}$$

where the eigenvector centrality scores correspond to the largest eigenvector of the graph adjacency matrix.

Betweenness centrality of node i measures the fraction of shortest paths that pass through a node in the network defined as:

$$C_B(i) = \frac{1}{(N-1)(N-2)} \sum_{s \neq i \neq r} \frac{\sigma_{s,r(i)}}{\sigma_{s,r}}, \tag{4}$$

where $\sigma_{s,r}$ is the shortest paths between source node s and target node r, $\sigma_{s,r(i)}$ is the number of shortest paths between source node s and target node r that pass through i, and N is the number of nodes in the network.

Closeness centrality measures the average shortest distance from one node to another in a network, defined as:

$$C_C(i) = \frac{1}{(N-1)} \frac{1}{\sum_r d(r,i)}, \tag{5}$$

where $d(r, i)$ is the distance between nodes i and r.

Both degree centrality and betweenness centrality will be normalized

over the different networks to make between-network comparison possible. Normalization is defined by:

$$\text{normal}(C(i)) = \frac{C(i) - \min(C)}{\max(C) - \min(C)}, \tag{6}$$

where $\max(C)$ is the maximum centrality value over all nodes in all networks in the simulation and $\min(C)$ is the minimum centrality value over all nodes in all networks in the simulation.

S3: Supplementary Figure, variance distribution

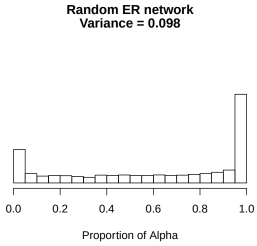

(a)

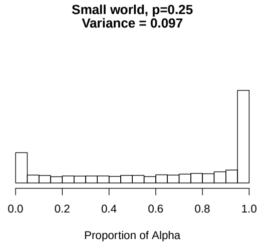

(b)

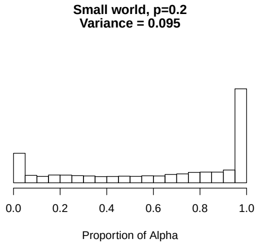

(c)

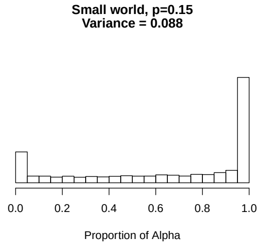

(d)

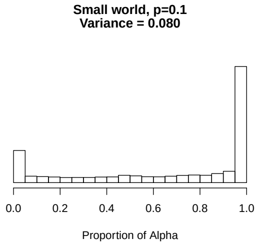

(e)

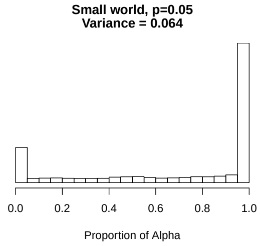

(f)

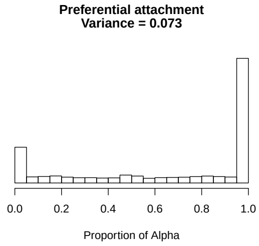

(g)

Figure 1: Variance of the proportion of times a node converges to the preferred equilibrium state, played on a network when the same initial distribution is played 100 times on the *same* network. A proportion of 1 indicates the node always converges to the preferred equilibrium state, 0 indicates the node never converges to the preferred equilibrium state.

S4: Supplementary Figure, Illustration of variable *Power*

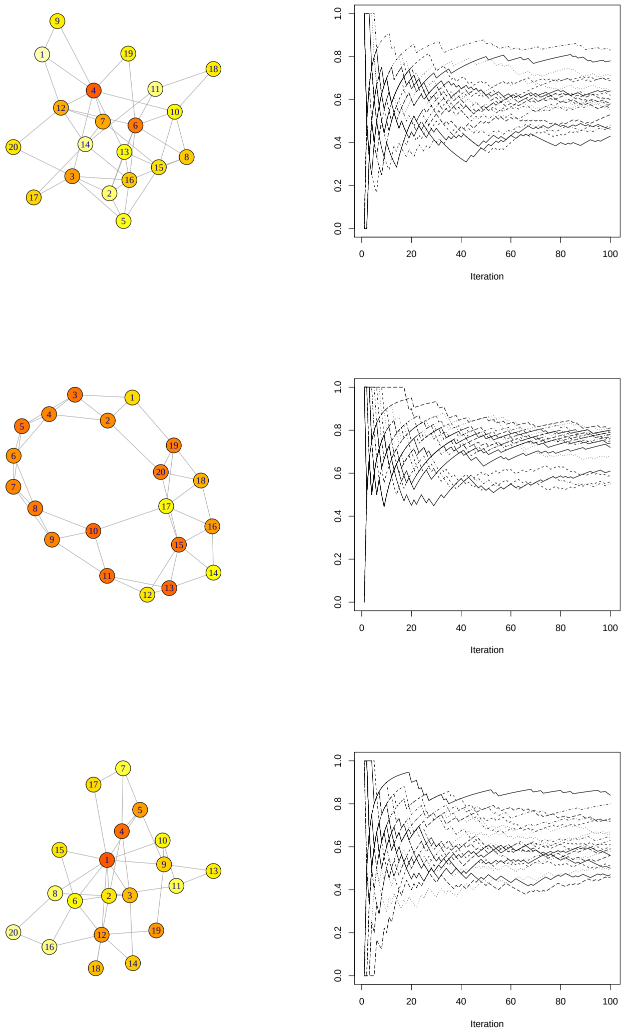

Figure 2: Left the network and right the estimate of node *Power* after playing different starting conditions on the same network. The lines indicate the proportion of times that the node ends in the preferred equilibrium.

S5: Supplementary Figure, Average heterogeneity over 200 replications (except for N = 640: 50 replications; N = 1280: 10 replications*), network density = 0.2

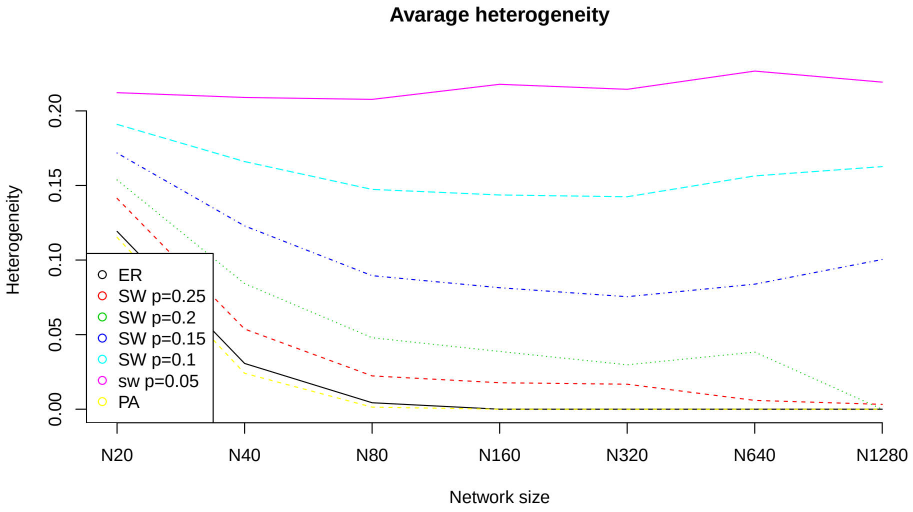

*The larger networks are computionally very intensive, therefore we used less replications for the network sizes N=640, N=1280. In addition, less replications are necessary because there is less variation between networks if network size is larger.

S6: Supplementary Figure, Average heterogeneity over 200 replications (except for N = 640: 50 replications; N = 1280: 10 replications*), network density = 0.1

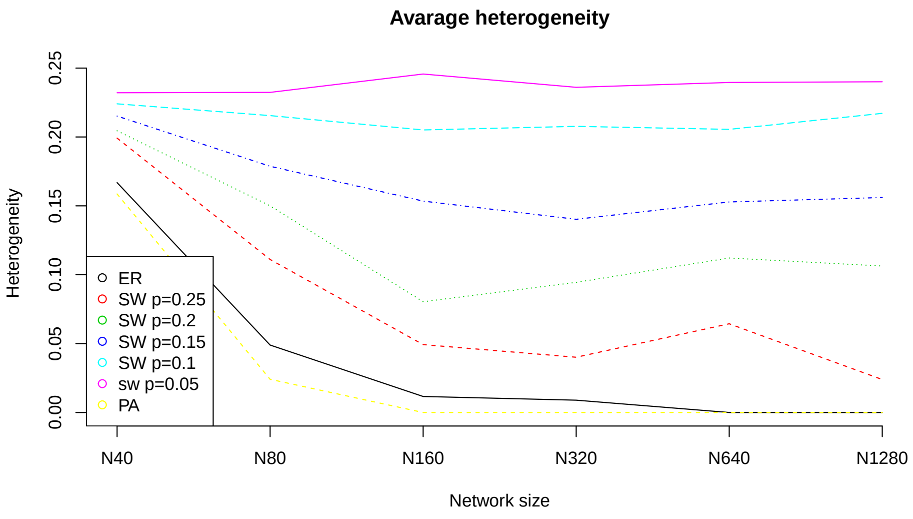

*The larger networks are computionally very intensive, therefore we used less replications for the network sizes N=640, N=1280. In addition, less replications are necessary because there is less variation between networks if network size is larger.

S7: Supplementary Figure, Average heterogeneity over 200 replications (except for N = 640: 50 replications; N = 1280: 10 replications*), network density = 0.3

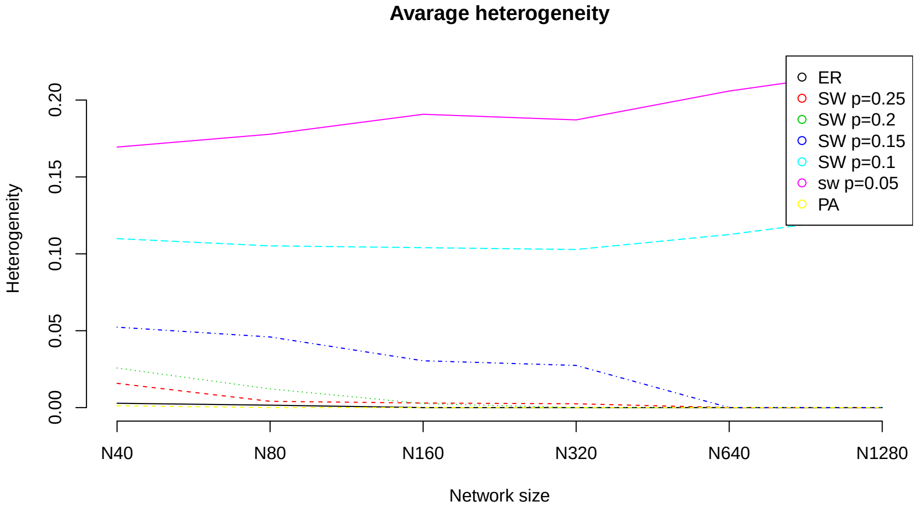

*The larger networks are computionally very intensive, therefore we used less replications for the network sizes N=640, N=1280. In addition, less replications are necessary because there is less variation between networks if network size is larger.

Supplementary Figure S8: Regression results with N=40

Table 2: Regression results, standardized, dependent variable *Power*, N=40.

	ER	SW $p = 0.25$	SW $p = 0.20$	SW $p = 0.15$	SW $p = 0.1$	SW $p = 0.05$	PA
Even		0.053	0.061	0.071	0.078	0.091	0.023
DegC	−0.006	0.210	0.215	0.190	0.116	0.065	0.316
EVC	0.153						
BetC	0.117						
Constant	0.424	0.409	0.421	0.466	0.517	0.554	0.452
N	20,000	4,000	4,000	4,000	4,000	4,000	20,000
R^2	0.361	0.391	0.370	0.354	0.367	0.417	0.532

**Even = variable indicating an even degree, EVC= Eigenvector centrality, BetC= Betweenness centrality, DegC = Degree centrality, ClosC= Closeness centrality. *Interaction in uncentered variables.*

Supplementary Figure S9: Regression results with N=80

Table 3: Regression results, standardized, dependent variable *Power*, N=80.

	ER	SW $p = 0.25$	SW $p = 0.20$	SW $p = 0.15$	SW $p = 0.1$	SW $p = 0.05$	PA
Even		0.053	0.010	0.014	0.053	0.091	
DegC	0.070	0.225	0.161	0.156	0.106	0.060	0.205
EVC	0.042						
BetC	0.064						
BetC:EVC	0.033						
Constant	0.420	0.423	0.444	0.463	0.507	0.548	0.465
N	20,000	4,000	4,000	4,000	4,000	4,000	20,000
R^2	0.272	0.211	0.182	0.161	0.158	0.152	0.401

**Even = variable indicating an even degree, EVC= Eigenvector centrality, BetC= Betweenness centrality, DegC = Degree centrality, ClosC= Closeness centrality. *Interaction in uncentered variables.*

S10: Supplementary Figure, Proportion of α in a network with S=0.7

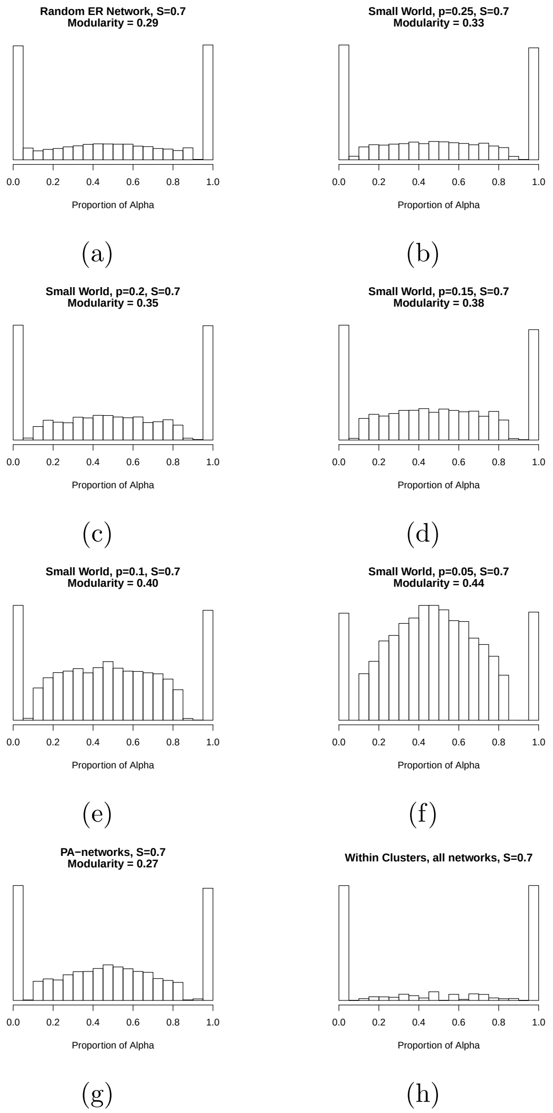

Figure 3: Proportion of α played in a network after convergence for S=0.7. (a) for random ER-Networks, (b) for small-world Networks with rewiring probability 0.25, (c) for small-world Networks with rewiring probability 0.2, (d) for small-world Networks with rewiring probability 0.15, (e) for small-world Networks with rewiring probability 0.1, (f) for small-world Networks with rewiring probability 0.05, (g) for PA-networks, (h) within clusters of all types of networks.

S11: Supplementary Figure, Proportion of α in a network with S=0.5

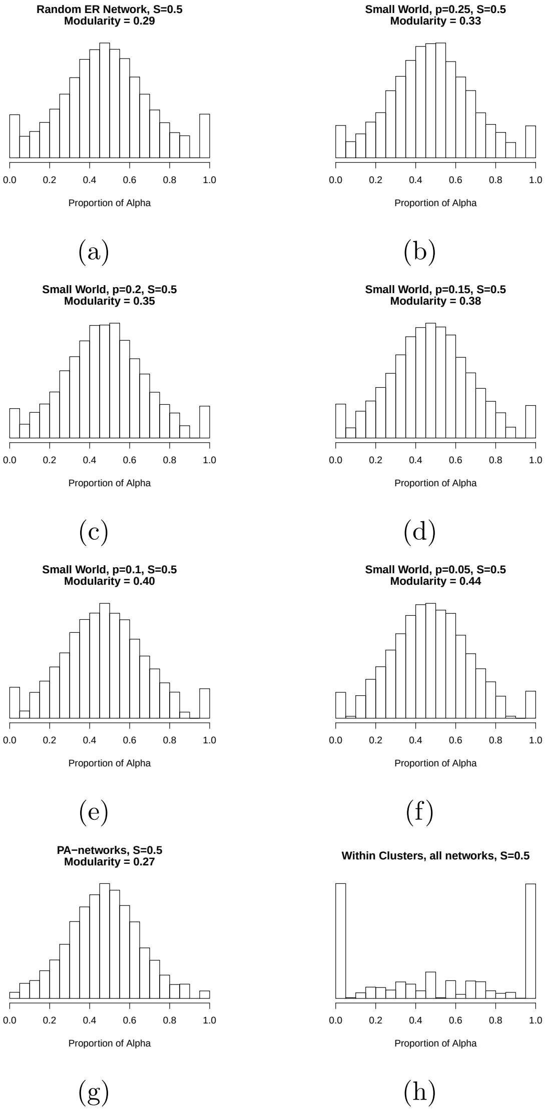

Figure 4: Proportion of α played in a network after convergence for S=0.5. (a) for random ER-Networks, (b) for small-world Networks with rewiring probability 0.25, (c) for small-world Networks with rewiring probability 0.2, (d) for small-world Networks with rewiring probability 0.15, (e) for small-world Networks with rewiring probability 0.1, (f) for small-world Networks with rewiring probability 0.05, (g) for PA-networks, (h) within clusters of all types of networks.

Supplementary Figure S12: Regression results with S=0.7

Table 4: Regression results, standardized, dependent variable *Power*.

	ER	SW $p = 0.25$	SW $p = 0.20$	SW $p = 0.15$	SW $p = 0.1$	SW $p = 0.05$	PA
Even	0.137	0.053	0.153	0.156	0.166	0.171	0.135
DegC	−0.172	0.272	0.265	0.203	0.152	0.113	0.489
EVC	−0.099						
BetC	0.358						
EVC:DegC	0.426						
Constant	0.543	0.460	0.469	0.487	0.513	0.526	0.516
N	20,000	4,000	4,000	4,000	4,000	4,000	20,000
R^2	0.546	0.614	0.632	0.627	0.652	0.650	0.522

**EVC= Eigenvector centrality, BetC= Betweenness centrality, DegC = Degree centrality, ClosC= Closeness centrality*
**Interaction in uncentered variables*

Supplementary Figure S13: Regression results with S=0.5

Table 5: Regression results, standardized, dependent variable *Power*

	ER	SW $p = 0.25$	SW $p = 0.20$	SW $p = 0.15$	SW $p = 0.1$	SW $p = 0.05$	PA
Even	−0.041	−0.087	−0.096	−0.102	−0.106	−0.102	−0.082
DegC	1.179	0.225		−0.012	0.020	0.105	0.240
ClosC	0.324						
BetC	0.211	0.246	0.270	0.202	0.141		
EVC	−0.132						
DegC:ClosC	−1.401						
DegC:EVC	0.034						
Constant	0.603	0.810	0.816	0.820	0.813	0.789	0.835
N	20,000	4,000	4,000	4,000	4,000	4,000	20,000
R^2	0.407	0.371	0.412	0.475	0.483	0.428	0.535

**EVC= Eigenvector centrality, BetC= Betweenness centrality, DegC = Degree centrality, ClosC= Closeness centrality*
**Interaction in uncentered variables*
